# Energy and Infrared Radiation Characteristics of the Sandstone Damage Evolution Process

**DOI:** 10.3390/ma16124342

**Published:** 2023-06-13

**Authors:** Hai Sun, Hong-Yan Zhu, Jie Han, Chun Fu, Mi-Mi Chen, Kun Wang

**Affiliations:** 1 School of Civil Engineering, Liaoning Petrochemical University, Fushun 113001, China; sunhai@lnpu.edu.cn (H.S.); fuchun@lnpu.edu.cn (C.F.); 19841341708@163.com (K.W.); 2 Key Lab of Petro-Chemical Special Building Materials, Fushun 113001, China

**Keywords:** rock failure, energy dissipation and release, infrared radiation, safety monitoring, microcrack

## Abstract

The mechanical characteristics and mechanisms of rock failure involve complex rock mass mechanics problems involving parameters such as energy concentration, storage, dissipation, and release. Therefore, it is important to select appropriate monitoring technologies to carry out relevant research. Fortunately, infrared thermal imaging monitoring technology has obvious advantages in the experimental study of rock failure processes and energy dissipation and release characteristics under load damage. Therefore, it is necessary to establish the theoretical relationship between the strain energy and infrared radiation information of sandstone and to reveal its fracture energy dissipation and disaster mechanism. In this study, an MTS electro-hydraulic servo press was used to carry out uniaxial loading experiments on sandstone. The characteristics of dissipated energy, elastic energy, and infrared radiation during the damage process of sandstone were studied using infrared thermal imaging technology. The results show that (1) the transition of sandstone loading from one stable state to another occurs in the form of an abrupt change. This sudden change is characterized by the simultaneous occurrence of elastic energy release, dissipative energy surging, and infrared radiation count (IRC) surging, and it has the characteristics of a short duration and large amplitude variation. (2) With the increase in the elastic energy variation, the surge in the IRC of sandstone samples presents three different development stages, namely fluctuation (stage Ⅰ), steady rise (stage Ⅱ), and rapid rise (stage Ⅲ). (3) The more obvious the surge in the IRC, the greater the degree of local damage of the sandstone and the greater the range of the corresponding elastic energy change (or dissipation energy change). (4) A method of sandstone microcrack location and propagation pattern recognition based on infrared thermal imaging technology is proposed. This method can dynamically generate the distribution nephograph of tension-shear microcracks of the bearing rock and accurately evaluate the real-time process of rock damage evolution. Finally, this study can provide a theoretical basis for rock stability, safety monitoring, and early warning.

## 1. Introduction

The fracturing of surrounding rock is the fundamental cause of coal pillar instability and mine water inrushing [1]. A large number of engineering measurements and tests have shown that the fracture mechanical characteristics and mechanisms of rock involve complex mechanical problems with respect to the rock mass such as energy concentration, storage, dissipation, and release [2,3]. Thus, rock fracture mechanics are the most valuable way to study coal rock failure and instability from an energy perspective. Numerous scholars have contributed to basic research on the energy dissipation and release laws of rock mass in the process of mechanical failure. Zhao et al. [4] proposed the minimum energy principle of rock mass dynamic failure. Xie et al. [5,6] concluded that the combined action of energy dissipation and energy release results in rock deformation and failure, and they proposed the strength loss criterion and global failure criterion based on energy dissipation and releasable strain energy, respectively. Liang et al. [7] found that energy dissipation caused rock damage, lithology deterioration, and loss of strength; however, the internal cause of sudden rock failure is energy release. Chen et al. [8] proposed a damage coefficient based on the energy evolution mechanism, and the mechanical properties and damage evolution process of rock were analyzed from the perspective of energy. Zhang [9] obtained the evolution and distribution rules of the elastic energy properties and dissipation energy of rock samples in the process of deformation and failure. Kong et al. [10] found that the formation of macroscopic cracks requires greater dissipation energy than the breeding and initiation of microcracks; further, the elastic energy drops sharply and the damage reaches the maximum when macroscopic cracks are formed. 

Considering the highly nonlinear process of rock failure, it is often difficult to invert the whole failure process effectively with only a single evaluation index. It is necessary to use a variety of monitoring equipment (stress–strain, acoustic emission, thermal infrared, etc.) to carry out research and tests with respect to the failure laws of stressed rocks at the laboratory scale and to identify reliable signals of failure precursors. Infrared thermal imaging is a nondestructive remote sensing monitoring technology [11,12,13] that has advantages in the study of rock failure processes and energy dissipation and release characteristics under load and has been widely used in rock failure warning systems. Shen et al. [14], Cao et al. [15], Wang et al. [16], and Lai et al. [17] proposed that spatial differentiation infrared thermal images, obvious turning of the average infrared radiation temperature curve, and the migration characteristics of abnormal regions of infrared radiation could be taken as the precursors of rock failure. Cai et al. [18] found that the existence of water promoted the release of heat energy. Aiming to reveal the law of infrared radiation of rock under an impact load, Zhou et al. [19] adopted the separated Hopkinson pressure bar (SHPB) system to carry out impact tests on sandstone under different strain rates and found that with the increase in the strain rate, the average infrared radiation temperature increment keeps increasing, and the relationship between them is a power function. Based on the characteristics of spatial differentiation in infrared thermal images before rock failure, Liu et al. [20], Ma et al. [21], Wu et al. [22], and Sun et al. [23] proposed the characteristic roughness index for infrared thermal images, differential infrared radiation variance, infrared temperature variation field, and infrared radiation count, respectively. These indexes quantitatively describe the evolution characteristics of the infrared radiation temperature field of rocks under load and provide a new idea for the infrared radiation analysis of rock disaster processes.

Few reports have associated the energy release law with infrared radiation characteristics. In this study, firstly, after analyzing the infrared radiation characteristics of rock and the release and dissipation characteristics of fracture energy under uniaxial compression, a quantitative relationship between infrared radiation parameters and rock strain energy parameters is established from the perspective of macroscopic energy conservation. Based on this relationship, the mechanical mechanism of the rock fracture degree is described so as to better reveal the fracture characteristics of rock. Secondly, a method based on infrared thermal imaging technology is proposed to identify the location and propagation mode of sandstone microcracks. This method can be used to dynamically generate the distribution cloud map of tension-shear microcracks of the bearing rock and accurately evaluate the real-time process of rock damage evolution. Finally, the results can provide a theoretical basis for rock stability, safety monitoring, and early warning.

## 2. Experimental Design

### 2.1. Experimental Samples

**Figure 1:**
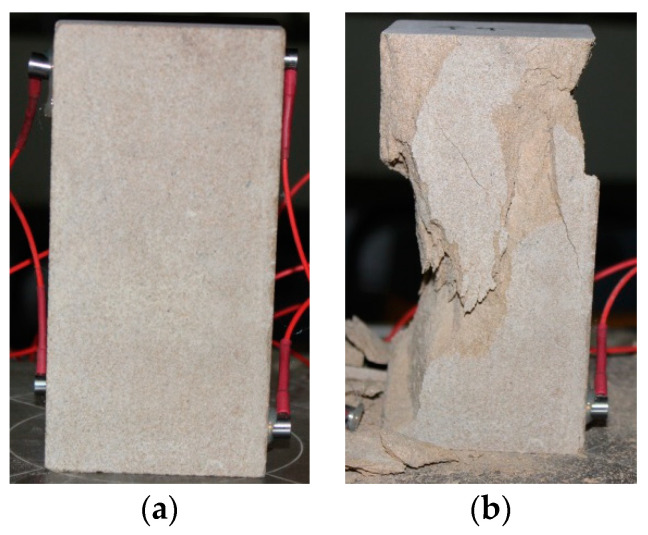
Photos of sandstone sample A_10_ before testing and after failure. (**a**) Before testing, (**b**) after failure.

Due to the uneven distribution of pores and microcracks, the internal structure of coal measure sandstone is quite different from that of other rocks. As a result, the dispersion of infrared observation experimental results with respect to such sandstone under uniaxial loading is large. In this study, sandstone samples with texture and uniformity were selected to ensure that the dispersion of the experimental results was small. The sandstone samples used in this study were from a quarry in the city of Jinan in Shandong Province and were extracted from a single block of rock. The sample size was 70 mm × 70 mm × 140 mm, and samples were numbered as A*_i_* (*i* = 1~13). All sandstone was processed in accordance with the requirements of the test procedures for rock physical and mechanical properties, as shown in Figure 1, and the mechanical properties of the tested sandstone samples are shown in Table 1. After processing, a grinding machine and sandpaper were used to carefully grind the two end-surfaces, and the longitudinal parallelism of both ends was not greater than 5 × 10^−5^ m. The sandstone was placed in the laboratory in advance so that the temperature of the sandstone was consistent with the ambient temperature of the laboratory.

### 2.2. Experimental System and Method

The uniaxial compression test of sandstone involved the equal displacement control method for loading, and the loading rate was controlled at 0.2 mm/min. A VarioCAM HD head 880 uncooled infrared thermal imager was used to collect the infrared radiation information of the sandstone during the whole process from loading to failure under uniaxial compression. The main parameters of the infrared thermal imager were as follows: thermal sensitivity > 0.02 °C; pixels, 1240 × 768; acquisition rate, 25 Fps/s; and measuring band, 7.5~14 µm. 

**Figure 2:**
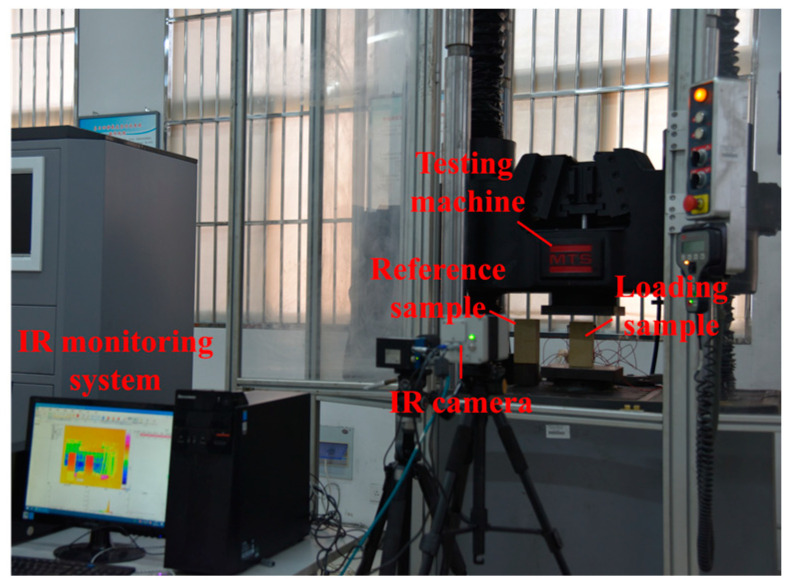
Infrared radiation monitoring system in the process of loading sandstone.

In the lead-up to the experiment, plastic films were arranged on the upper and lower contact surfaces between the sandstone and the loading machine to reduce the friction and heat conduction effect between the sandstone and the loading head during the experiment. The advantage of this was that the end effect could be reduced but the mechanical properties of sandstone did not changed. The reference sample was placed parallel to the left of the loaded sandstone, but the reference sandstone was not loaded, as shown in Figure 2. No one was allowed to move around during the experiment, and the windows, curtains, and all radiation-generating lighting sources of the laboratory were closed [24]. 

## 3. Experimental Results and Analysis

### 3.1. Variation Characteristics of Strain Energy in Sandstone Fracture Process

Assuming that there was no energy exchange between the sandstone and the external environment during the loading process, according to the first law of thermodynamics [5,6]:

(1) $$U=U^{e}+U^{d}$$

where *U* is the total energy input by the loading machine, $U^{e}$ is the elastic energy stored in the sandstone, and $U^{d}$ is the dissipation energy of the sandstone. Therefore, the total energy absorbed under uniaxial loading is as follows [5,6]:

(2) $$U={\int \sigma }_{1}d\varepsilon_{1}=\sum_{i=1}^{n}\frac{1}{2}(\varepsilon_{1,i+1}-\varepsilon_{1})(\sigma_{1,i+1}+\sigma_{1})$$

where $\sigma_{1}$ is stress and $\varepsilon_{1}$ is strain. 

The elastic energy can be expressed as follows [5,6]:

(3) $$U^{e}=\frac{1}{2}\sigma_{1}\varepsilon_{1}^{e}=\frac{\sigma_{1}^{2}}{2E_{u}}\approx \frac{\sigma_{1}^{2}}{2E_{0}}$$

where $E_{0}$ is the initial elastic modulus. 

Therefore, the dissipation energy is as follows:

(4) $$U^{d}=U-\frac{\sigma_{1}^{2}}{2E_{0}}$$

**Figure 3:**
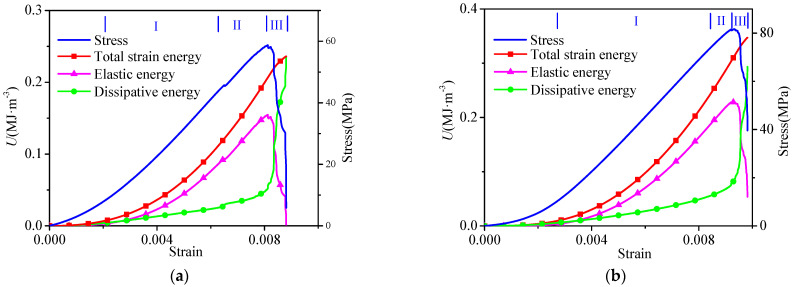

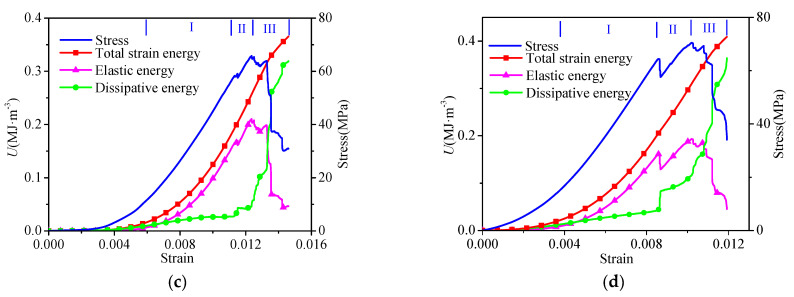
Relationship between stress, energy, and strain of sandstone under uniaxial loading. (**a**) A_3_ sandstone, (**b**) A_5_ sandstone, (**c**) A_7_ sandstone, and (**d**) A_10_ sandstone.

Hence, the energy value of the sandstone in the loading process could be calculated based on the abovementioned energy calculation equations. The energy curve variation characteristics corresponding to the compaction stage, linear elastic deformation stage, plastic deformation stage, and post-peak stage of all the sandstone samples had similar characteristics, as shown in Figure 3. From Figure 3, it can be seen that in the linear elastic stage (I), most of the work performed by external forces was converted into releasable elastic energy and stored in the sandstone, so the increase rate of elastic energy was faster, the slope of the curve was larger, and the slope of the dissipation energy curve was approximately equal. In Figure 3, it can also be seen that the total strain energy and elastic energy increased almost in the same trend, and the increase rates of both were greater than that of the dissipation energy.

In the plastic deformation stage (II) shown in Figure 3, plastic deformation occurred at the same time as elastic deformation, and the process was also accompanied by the initiation and development of microcracks, so the releasable elastic energy and dissipation energy increased gradually. This stage was the key stage for new crack breeding, formation, and propagation in the sandstone; therefore, the total input energy was mainly transformed into the dissipation energy driving the failure of the sandstone, which was manifested as the growth rate of the dissipation energy and the slope of the dissipated energy curve increasing. After the formation of cracks in the sandstone, the material deteriorated and the energy storage capacity decreased, so the elastic energy tended to slow down. When the total stress–strain curve of some sandstone samples reached the peak stress, there was a slight drop in the stress, the stored elastic energy was released quickly with a small amplitude, and there was a small surge increase in energy dissipation (as shown in Figure 3c,d).

In the post-peak stage (III) shown in Figure 3, the sandstone required more energy to drive the crack coalescence, so the dissipation energy increased sharply, and the slope of the dissipation energy curve became larger. After the formation of large-scale cracks, the sandstone lost the ability to store energy, and the releasable elastic energy stored in the sandstone was released in large quantities and converted into dissipation energy. Hence, in Figure 3, the elastic energy suddenly decreased, and the dissipation energy and the growth rate gradually increased.

### 3.2. Infrared Radiation Characteristics of Sandstone Fracture Process

#### 3.2.1. Infrared Radiation Index

The infrared radiation information of bearing sandstone is composed of effective signals (microcracks are generated resulting in changes in infrared radiation) and noise signals (environmental temperature changes and uncooled infrared focal plane array response drift with time, resulting in changes in infrared radiation), and the infrared radiation variation amplitude of the reference sandstone is only due to noise signals [25,26]. In order to eliminate the interference of noise signals in relation to the effective signals of the bearing sandstone in this study, the threshold value of the infrared radiation temperature matrix of the bearing sandstone at the corresponding time was determined by the maximum value in the infrared radiation temperature matrix of each frame of the reference sandstone. When the value in the infrared radiation temperature matrix of the bearing sandstone was greater than the threshold value, it was regarded as the infrared radiation temperature change caused by damage to the bearing sandstone, and the temperature value is known as the infrared radiation sudden change temperature. The IRC is obtained by obtaining statistics on the infrared radiation temperature change matrix of sandstone that are larger than the threshold value [23]. The physical significance of the IRC is the number of cracks produced by sandstone at a certain time in the process of the damage evolution of sandstone. The greater the IRC at a certain time, the more cracks produced inside the sandstone at that time, and the more serious the damage. 

#### 3.2.2. Characteristics of Infrared Radiation Time Sequence Change

**Figure 4:**
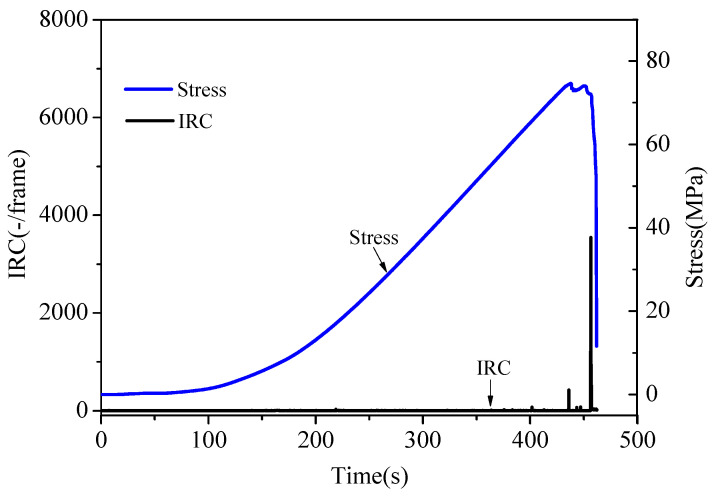
Stress and IRC varied with time for sandstone sample A_2_.

The relationship between the IRC and stress is shown in Figure 4. The periodic variation characteristics of the IRC of all the sandstone samples were similar. Due to limited space, only sandstone A_2_ is given as an example. When the loading times were 436.0 s (where the stress was 92.2% of the peak stress) and 455.8 s (where the stress was 94.2% of the peak stress), the IRC had a pulse surge with a sudden stress drop in the loading curve (the IRC immediately reverted to its pre-surge state). 

#### 3.2.3. Spatial Variation Characteristics of Infrared Radiation

**Figure 5:**
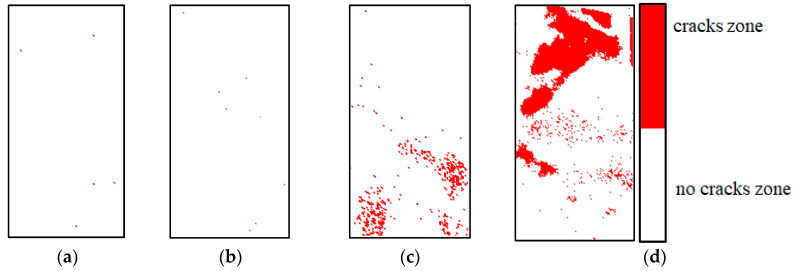
Cloud maps of IRC spatial distribution of sandstone sample A_2_. (**a**) Compaction stage, (**b**) elastic stage, (**c**) moment of first sudden change in IRC, and (**d**) moment of second sudden change in IRC.

In order to study the spatial distribution characteristics of infrared radiation in the loading process of sandstone, a cloud map of the IRC spatial distribution of sandstone A_2_ in the compaction stage, elastic stage, plastic stage, and the moment of IRC surge is shown in Figure 5a–d, respectively. An IRC spatial distribution cloud map can show the location of new cracks in sandstone at a certain time. The higher the IRC, the more cracks there are in the sandstone and the more serious the damage is. Before the IRC surge (as shown in Figure 5a,b), cracks were disordered in the spatial distribution cloud map of the IRC, and the number of cracks was at a low scale. Only when the IRC mutated were the cracks mainly distributed in a certain area in the spatial distribution cloud map of the IRC. According to the moment of the IRC surge in the sandstone, the spatial distribution map of the IRC at the corresponding time could be obtained, and the damaged and broken areas of the sandstone could be accurately located. 

### 3.3. Correlation between Infrared Radiation and Strain Energy in Sandstone Loading Process

**Figure 6:**
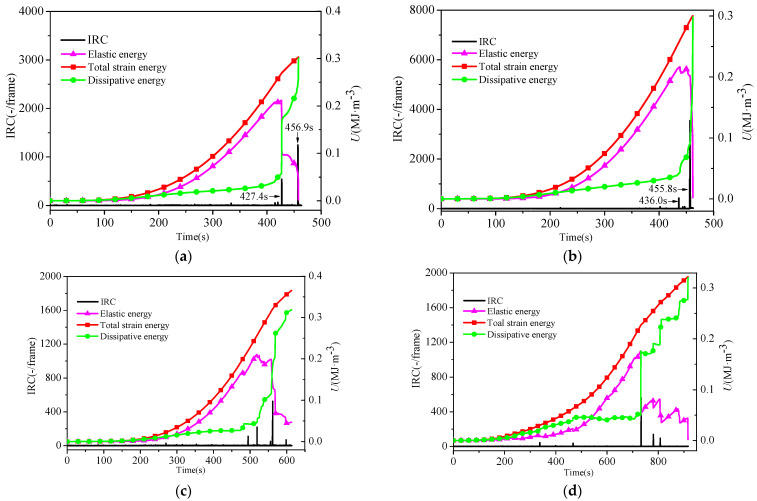
Relationship between sandstone IRC, elastic energy, dissipation energy, and time. (**a**) A_1_ sandstone, (**b**) A_2_ sandstone, (**c**) A_8_ sandstone, and (**d**) A_10_ sandstone.

The relationship between the IRC and strain energy of the loaded sandstone over time is shown in Figure 6. In the compaction stage and elastic stage, the IRC did not show obvious surge characteristics with the accumulation of elastic energy and dissipation energy. In the plastic stage and failure stage, the elastic energy release, dissipation energy surge, and IRC surge occurred synchronously in all the sandstone samples. Taking sandstone A_1_ as an example, the IRC surge occurred synchronously at 427.4 s and 456.9 s during the elastic energy release and dissipation energy surge, as shown in Figure 6a. In the plastic stage, the dissipation energy began to rise rapidly after the first IRC surge, and at the end of loading, the dissipation energy reached its maximum. The above phenomena indicate that the transition of the loaded sandstone from a stable state to another stable state occurred in the form of a sudden change, which was specifically manifested as the simultaneous occurrence of the elastic energy release, the surge in dissipated energy, and the surge in the IRC. This phenomenon represents a transient change in state with the characteristics of a short period and large amplitude.

### 3.4. Quantitative Relationship between Infrared Radiation and Strain Energy in Sandstone Stress Drop

When the stress drop occurred in the sandstone, its energy was released and dissipated at the same time as the internal structure was damaged. Meanwhile, the IRC mutated simultaneously. As a result, there was good correspondence between the elastic energy release, dissipation energy surge, and IRC surge of the sandstone. There must be a quantitative relationship between the strain energy change information and IRC surge information. For the sake of exploring the quantitative relationship between the variation in strain energy and the surge of infrared radiation during the generation of the stress drop in the sandstone, three variation indexes based on elastic energy, dissipated energy, and IRC were proposed in this study, and the corresponding three indexes of each stress drop process were calculated and counted. 

The variation in elastic energy ($\Delta U^{e}$) is the difference between the highest energy ($U_{a}^{e}$) and the lowest energy ($U_{b}^{e}$) of the elastic energy during the generation of the stress drop in sandstone:

(5) $$\Delta U^{e}=U_{a}^{e}-U_{b}^{e}$$

The variation in dissipation energy ($\Delta U^{d}$) is the difference between the highest energy value ($U_{a}^{d}$) and the lowest energy value ($U_{b}^{d}$) of the dissipation energy during the generation of the stress drop in sandstone:

(6) $$\Delta U^{d}=U_{a}^{d}-U_{b}^{d}$$

The variation in IRC (ΔIRC) refers to the absolute difference between the highest value (${\mathrm{IRC}}_{a}$) and the lowest value (${\mathrm{IRC}}_{b}$) of the IRC at the corresponding moment when elastic energy release occurs in sandstone:

(7) $$\Delta \mathrm{IRC}={\mathrm{IRC}}_{a}-{\mathrm{IRC}}_{b}$$

The $\Delta U^{e}$, $\Delta U^{d}$, and ΔIRC values of the sandstone samples are shown in Table 2. The phenomena of elastic energy release, dissipation energy surge, and IRC surge occurred simultaneously 21 times in 13 sandstone samples. Among them, eight samples showed one instance of elastic energy release, surge in dissipation energy, and synchronous IRC surge, while multiple elastic energy releases, dissipated energy surges, and IRC surges synchronously occurred in the other five samples.

The variation in the elastic energy of the sandstone samples ranged from 0.002 to 0.449 MJ/m^3^ (average was 0.083 MJ/m^3^), and the variation in dissipation energy ranged from 0.003 to 0.561 MJ/m^3^ (average was 0.092 MJ/m^3^); the variation in dissipation energy was slightly higher than that of elastic energy. The stress ratios for the above phenomena ranged from 34.63% to 100% (the average was 91%).

**Figure 7:**
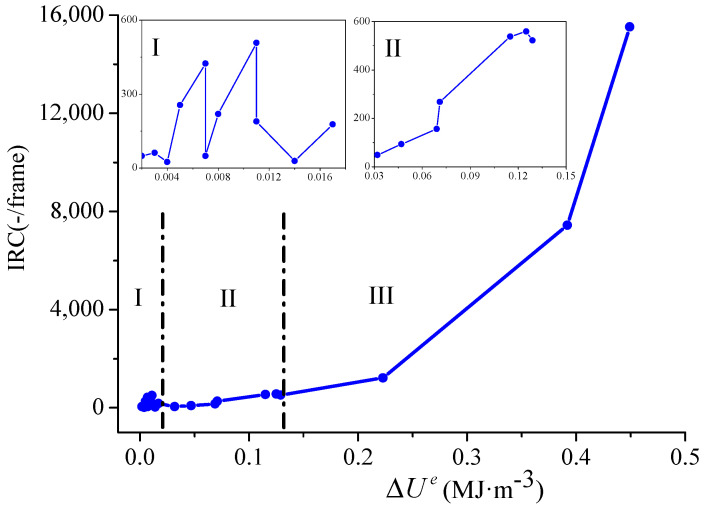
Relationship between IRC mutation and the variation in elastic energy.

**Figure 8:**
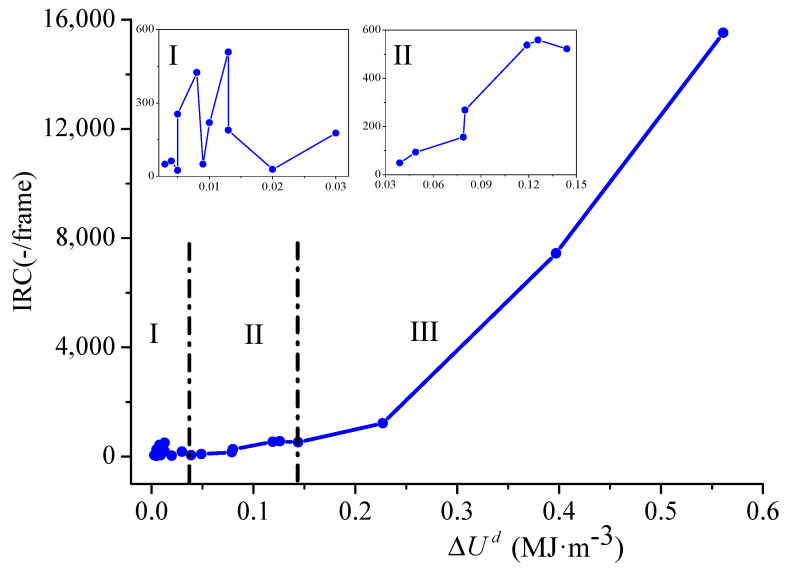
Relationship between IRC mutation and the variation in dissipated energy.

Figure 7 and Figure 8 show the relationship between ΔIRC and $\Delta U^{e}$, and ΔIRC and $\Delta U^{d}$, for all the sandstone samples, respectively. The trends in the two figures were basically the same; therefore, only the diagram of the relationship between ΔIRC and $\Delta U^{e}$ is illustrated here. The sandstone samples showed three development stages with different increasing trends, i.e., stage Ⅰ, fluctuation; stage Ⅱ, steady rise; and stage Ⅲ, rapid rise (as shown in Figure 7). With the increase in $\Delta U^{e}$, ΔIRC had three different development stages, with stage Ⅰ being fluctuation, stage Ⅱ being a steady rise, and stage Ⅲ being a rapid ascent, as shown in Figure 7.

Stage Ⅰ, fluctuation: In eleven cases (about 52.4% of the total), ΔIRC tended to oscillate with the growth in $\Delta U^{e}$. In this stage, $\Delta U^{e}$ ranged from 0.002 to 0.017 MJ/m^3^ (average was 0.008 MJ/m^3^), and ΔIRC ranged from 24 to 508 (average was 180.4). 

Stage Ⅱ, steady rise: In seven cases (about 33.3% of the total), ΔIRC tended to rise steadily with the growth in $\Delta U^{e}$. In this stage, $\Delta U^{e}$ ranged from 0.032 to 0.129 MJ/m^3^ (average was 0.084 MJ/m^3^), and ΔIRC ranged from 49 to 559 (average was 312.3). 

Stage Ⅲ, rapid ascent: In three cases (about 14.3% of the total), ΔIRC tended to ascend rapidly with the growth in $\Delta U^{e}$. In this stage, $\Delta U^{e}$ ranged from 0.223 to 0.449 MJ/m^3^ (average was 0.355 MJ/m^3^), and ΔIRC ranged from 1223 to 15,519 (average was 8061.3). In summary, ΔIRC increased with the growth in $\Delta U^{e}$ or $\Delta U^{d}$. These results indicate that the magnitude of the stress drop in the sandstone depended on the local damage degree of the sandstone samples, namely, the more obvious the IRC surge, the greater the local damage degree of the sandstone and the larger the corresponding amplitude.

## 4. Discussion 

### 4.1. Sensitivity Analysis of Infrared Radiation Index

**Figure 9:**
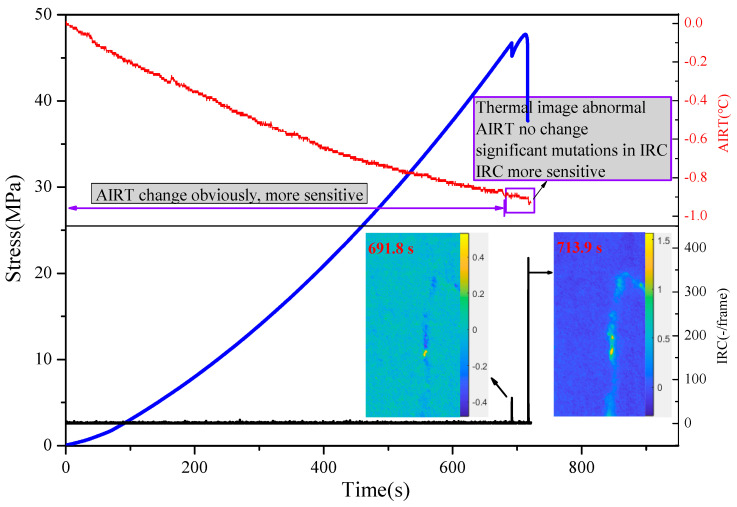
Sensitivity of sandstone sample A_7_ to infrared radiation information.

Figure 9 shows the variation law of the average infrared radiation temperature (AIRT) and IRC of sandstone A_7_ after de-noising during the loading process. The AIRT can directly reflect the overall infrared radiation intensity of the bearing sandstone surface, which is an important feature reflecting the change in infrared radiation. Therefore, the average infrared radiation temperature can be used as a quantitative analysis index to reflect the change characteristics of infrared radiation in the bearing sandstone. 

The physical significance of the IRC is that the number of cracks in sandstone at a certain time in the process of damage evolution can be obtained based on infrared thermal imaging technology. As can be seen from Figure 9, the variation characteristics of the AIRT and IRC indexes were different in the different loading stages of the sandstone. From the initial loading to the first stress drop, the AIRT of sandstone A_7_ had an obvious downward trend, from the initial value of 0 °C to −0.88 °C. The IRC showed no significant change. This indicates that at this stage, the AIRT index was more sensitive to the change in the sandstone state than the IRC. During the time from the first stress drop to the final failure of the sandstone, the thermal image of sandstone A_7_ showed obvious anomalies at 691.8 s and 713.9 s, the change trend of the AIRT was slow and had no obvious change characteristics, and the IRC synchronization showed a significant mutation.

The reasons for the difference and asynchronism between the AIRT anomaly and infrared thermal image anomaly are as follows. First, the generation of infrared radiation information is closely related to the failure form of sandstone. When there are thermal effects with opposite trends on the surface of a sandstone failure (i.e., shear cracks have a heating effect and tension cracks have a cooling effect [20]), such opposite thermal effects offset each other, resulting in insignificant changes in the AIRT [20]. Second, when the sandstone was broken, the infrared thermal image only showed anomalies in small areas, and the overall infrared radiation temperature of the sandstone surface changed very little.

In contrast, the AIRT had a better response to both the compaction stage and elastic stage of the sandstone, while the IRC had a better response to the generation of the stress drop in the plastic stage and failure stage of the sandstone. The amplitude of the IRC changed little before the fracture (stress drop) of the sandstone, and abrupt changes occurred with the stress drop near the moment of sandstone failure. Moreover, the spatial distribution map of the IRC reflected the evolution and differentiation characteristics of the sandstone surface in the process of fracture and failure. As a consequence, the IRC has more advantages in terms of the identification of damage information and infrared radiation precursors of sandstone, and this makes it easier to capture the precursors of the failure of bearing sandstone. It is suitable for use as an indicator to find the precursors of the failure of bearing sandstone and establish the index of the quantitative relationship between the damage information of sandstone and the infrared radiation information.

### 4.2. Identification of Location and Propagation Pattern of Sandstone Microcracks

**Figure 10:**
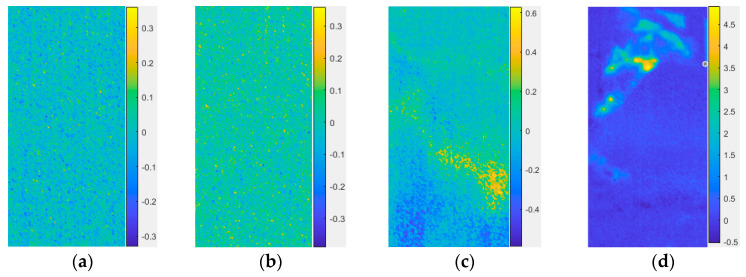
Differential infrared thermal image of sandstone A_2_: (**a**) compaction stage, (**b**) elastic stage, (**c**) moment of first sudden change in IRC, and (**d**) moment of second sudden change in IRC.

There are great uncertainties in the location and propagation pattern recognition of sandstone microcracks. The variation amplitude of the infrared radiation temperature in sandstone is closely related to the propagation mode of the microcracks. Namely, shear cracks have a heating effect and tension cracks have a cooling effect [20]. If the points where the temperature rises and cools can be screened from the infrared radiation temperature field, the location and propagation mode of sandstone microcracks can be identified. Previous studies have shown that the region above 0 °C in differential infrared thermography represents the warming region, while the region below 0 °C represents the cooling region [21]. It is difficult to distinguish the local infrared radiation temperature variation in differential infrared thermal image sequences. Taking sandstone A_2_ as an example, the differential infrared thermal image at the corresponding moment in Figure 5 is shown in Figure 10, and the rising and cooling zones are difficult to identify in Figure 10a,b,d.

**Figure 11:**
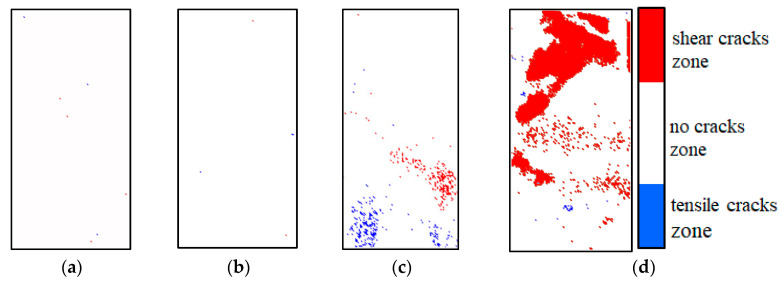
Distribution of tensile and shear cracks in sandstone A_2_: (**a**) compaction stage, (**b**) elastic stage, (**c**) moment of first sudden change in IRC, and (**d**) moment of second sudden change in IRC.

Aiming to solve the above problems, a method is presented in this paper to identify the location and propagation mode of sandstone microcracks. Firstly, the location information of the cracks in the spatial distribution cloud map of the sandstone IRC was determined, and then the corresponding positions of these cracks in the differential thermal map were determined as warming points or cooling points. Warming points are shown in red in the spatial distribution cloud map of the sandstone IRC (representing a shear crack). Cooling points are shown in blue (representing a tensile crack). According to the above mentioned method, the spatial distribution diagram of the sandstone tensile and shear cracks at the corresponding moment in Figure 5 is shown in Figure 11. The distribution of the abnormal region in the spatial distribution map of the tensile crack based on the IRC was basically the same as that of the differential infrared thermal image sequence map. The spatial distribution cloud map of tensile cracking based on the IRC could easily and intuitively identify the shear crack region and tension crack region on the surface of sandstone, which could more clearly reflect the information of damage evolution and fracturing failure of sandstone and could accurately locate the damage and fracturing regions of sandstone. 

### 4.3. Engineering Value and Practical Significance

The experimental results showed that when a stress drop occurred in the sandstone, the release and dissipation of strain energy were generated synchronously with the IRC mutation. The spatial distribution map of the IRC at the corresponding time can be drawn to accurately locate the damaged and broken area of sandstone, which is important for the monitoring and early warning of surrounding rock instability in mines, tunnels, and other geotechnical projects. The essence of the instability failure of engineering rock mass is the process of crack development until the loss of the peak bearing capacity, such as coal pillar instability, roadway surrounding rock instability, slope instability, etc. The rock mass in this study experienced crack closure, elastic deformation, stable crack development, unstable crack propagation, and a post-peak stage under the action of stress. In the phase of unstable crack growth, internal macroscopic cracks began to form, combine, and expand. The rock mass was irreversibly damaged, and its inherent strength was reduced. At this time, a large amount of elastic energy was dissipated and converted into other forms of energy (including infrared energy), which caused the temperature of the infrared radiation temperature field to rise, and the IRC underwent obvious mutations. Therefore, in practical application, thermal images can be used for the real-time monitoring of engineering rock mass, such as coal pillars, roadway-surrounding rock, and side slopes. The significant abrupt change in the IRC indicated an abrupt change in the rock fracture state (occurring in an unstable extension of the rock). It should be noted that the time interval between the IRC mutation and peak stress in the experiment was relatively short. This means that if a significant IRC mutation is observed, then the rock is close to instability. Therefore, corresponding necessary measures should be taken in time in actual projects, such as strengthening supports and evacuating nearby workers and equipment so as to avoid unnecessary losses. 

## 5. Conclusions

The infrared radiation information of rock is related to its energy dissipation and release, which can reflect the deformation and failure processes of rock. Previous studies did not correlate the law of strain energy release with infrared radiation characteristics. In this study, the quantitative relationship between infrared radiation parameters and rock strain energy parameters was established from the perspective of macroscopic energy conservation, and based on this relationship, the mechanical mechanism of the rock fracture degree was described. Furthermore, a method based on infrared thermal imaging was proposed to accurately evaluate the real-time process of rock damage evolution. The results provided a theoretical basis for rock stability, safety monitoring, and early warning. The conclusions are as follows:

(1) The relationship between infrared radiation parameters and rock strain energy parameters in the process of sandstone stress was determined. The simultaneous phenomenon of elastic energy release, dissipated energy surge, and infrared radiation count surge occurred when a stress drop occurred in all the sandstone samples. Among these, 61.5% of the sandstone samples exhibited the simultaneous occurrence of elastic energy release, surging of dissipated energy, and surging of the infrared radiation count, while 38.5% of the samples exhibited multiple simultaneous occurrences of elastic energy release, surging of dissipated energy, and surging of the infrared radiation count.

(2) Quantitative analysis indexes of the variation in elastic energy and mutation of the infrared radiation count were proposed, which can quantify the relationship between infrared radiation parameters and rock strain energy parameters. With the increase in the elastic energy variation, the mutation of the infrared radiation count of the sandstone samples showed three different development stages: stage Ⅰ, fluctuation; stage Ⅱ, steady rise; and stage Ⅲ, rapid ascent. In general, the greater the mutation of the infrared radiation count, the greater the local damage degree of the sandstone, and the greater the amplitude of the corresponding elastic change (or dissipated energy change).

(3) Based on the relationship between the infrared radiation parameters and the rock strain energy parameters, the mechanical mechanisms of the rock fracture degree were described, and the fracture characteristics of the rock mass were better revealed. Sandstone loading is the process of a transition from a stable state to another stable state, and this process takes place in the form of mutation. This sudden change is a transient state, which is characterized by a short period and large amplitude. 

(4) A method based on infrared thermal imaging was proposed to identify the location and propagation mode of sandstone microcracks, which can dynamically generate a distribution cloud map of tensile and shear microcracks of bearing rock, accurately evaluate the real-time process of rock damage evolution, easily and intuitively identify the shear crack regions and tension crack regions of the surface of sandstone, more clearly reflect the information of sandstone damage evolution and fracturing failure, and accurately locate regions of sandstone damage and fracturing.

However, this study was completed under uniaxial loading conditions, which do not fully reflect the real influence of mining modes and engineering disturbances. In future research, the quantitative relationship between the infrared radiation parameters and strain energy parameters of sandstone under different stress conditions such as shear, biaxial, and triaxial stress can be considered. In addition, consideration should be paid to the fact that in mine engineering research, sandstone is often in different water-bearing states. The presence of water can not only change the characteristics of the variation in sandstone strain but can also affect the infrared radiation characteristics of sandstone under stress. Subsequent studies should also fully consider the influence of water on the quantitative relationship between the infrared radiation parameters and the strain energy parameters of sandstone.

## Figures and Tables

**Table 1:** Mechanical properties of the tested samples of sandstone.

Specimen No.	Peak Stress (MPa)	Elastic Modulus (10^4^ MPa)
A_1_	71.27	1.2
A_2_	74.62	1.3
A_3_	58.92	1.4
A_4_	43.64	1.1
A_5_	81.80	1.1
A_6_	53.08	1.1
A_7_	47.77	1.0
A_8_	58.56	0.4
A_9_	62.94	1.3
A_10_	70.52	1.3
A_11_	96.31	1.4
A_12_	106.56	1.4
A_13_	65.69	1.5

**Table 2:** Variation in infrared radiation and strain energy in sandstone samples at the time of the stress drop.

Sample No.	$\Delta U^{e}$	$\Delta U^{d}$	ΔIRC	Stress Drop/Peak Stress
A_1_	0.002	0.003	49	99.09%
	0.003	0.004	62	99.67%
	0.115	0.119	538	100.00%
A_2_	0.007	0.008	424	100.00%
A_3_	0.223	0.227	1223	97.76%
A_4_	0.071	0.080	268	100.00%
	0.004	0.005	24	43.76%
	0.007	0.009	49	34.63%
A_5_	0.005	0.005	255	100.00%
A_6_	0.011	0.013	508	100.00%
A_7_	0.008	0.010	219	100.00%
	0.129	0.144	522	96.98%
A_8_	0.125	0.126	559	100.00%
	0.047	0.049	94	68.01%
A_9_	0.011	0.013	189	100.00%
A_10_	0.032	0.039	49	91.29%
	0.014	0.030	28	99.89%
	0.069	0.079	156	88.51%
A_11_	0.017	0.020	177	91.33%
A_12_	0.392	0.397	7442	100.00%
A_13_	0.449	0.561	15519	100.00%
Average	0.083	0.092	1350.19	91%

## Data Availability

The data presented in this study are available upon request from the corresponding author.

## Nomenclature

IRC	Infrared radiation counts
$\Delta U^{e}$	Variation in elastic energy
$\Delta U^{d}$	Variation in dissipation energy
ΔIRC	Variation in IRC
AIRT	Average infrared radiation temperature
